# Impact of different CBCT imaging monitor units, reconstruction slice thicknesses, and planning CT slice thicknesses on the positioning accuracy of a MV‐CBCT system in head‐and‐neck patients

**DOI:** 10.1120/jacmp.v13i5.3766

**Published:** 2012-09-06

**Authors:** Ming X. Jia, Xu Zhang, Na Li, Cheng B. Han

**Affiliations:** ^1^ Department of Radiation Oncology Shengjing Hospital of China Medial University Shenyang 110022 China

**Keywords:** MV‐CBCT, positioning accuracy, head‐and‐neck patients

## Abstract

The purpose of this study was to investigate the impact of different CBCT imaging monitor units (MUs), reconstruction slice thicknesses, and planning CT slice thicknesses on the positioning accuracy of a megavoltage cone‐beam computed tomography (MV‐CBCT) system in image‐guided radiation therapy (IGRT) in head‐and‐neck patients. The MV‐CBCT system was a Siemens MVision, a commercial system integrated into the Siemens ONCOR linear accelerator. The positioning accuracy of the MV‐CBCT system was determined using an anthropomorphic phantom while varying the MV‐CBCT imaging MU, reconstruction slice thickness, and planning CT slice thickness. A total of 240 CBCT images from six head‐and‐neck patients who underwent intensity‐modulated radiotherapy (IMRT) treatment were acquired and reconstructed using different MV‐CBCT scanning protocols. The interfractional setup errors of the patients were retrospectively analyzed for different imaging MUs, reconstruction slice thicknesses, and planning CT slice thicknesses. Using the anthropomorphic phantom, the largest measured mean deviation component and standard deviation of the MVision in 3D directions were 1.3 and 1.0 mm, respectively, for different CBCT imaging MUs, reconstruction slice thicknesses, and planning CT slice thicknesses. The largest setup group system error (M), system error (∑), and random error (σ) from six head‐and‐neck patients were 0.6, 1.2, and 1.7 mm, respectively. No significant difference was found in the positioning accuracy of the MV‐CBCT system between the 5 and 8 MUs, and between the 1 and 3 mm reconstruction slice thicknesses. A thin planning CT slice thickness may achieve higher positioning precision using the phantom measurement, but no significant difference was found in clinical setup precision between the 1 and 3 mm planning CT slice thicknesses.

PACS number: 87.55 ne

## I. INTRODUCTION

Three‐dimensional conformal radiotherapy (3D‐CRT) and intensity‐modulated radiation therapy (IMRT) have been widely used in the treatment of cancer. These techniques require greater precision in treatment setup and delivery than conventional techniques if the dose delivered to the target area must be assured. The importance of accurate daily setup and treatment delivery has been clinically demonstrated for advanced radiation therapy techniques.^1^, ^6^ Thus, verification of the patient treatment position is a crucial aspect of radiation therapy.

Imaging techniques have long played a key role in assuring the accuracy of radiation therapy treatment. In‐room 3D imaging systems for image‐guided radiation therapy (IGRT) have been widely used in radiation oncology as the tool of choice for patient setup verification.^7^, ^14^ The greatest advantages sought from the development of these new technologies over previous two‐dimensional (2D) imaging approaches are an increased positioning accuracy using volumetric information, and the possibility of observing not only bony anatomy but also specific soft‐tissue structures to assess patient setup and anatomical changes that occur during the course of therapy.^15^, ^17^

Megavoltage cone‐beam computed tomography (MV‐CBCT) is one such approach in the IGRT procedure using commercially available IGRT systems.^18^, ^20^ This technique uses mega‐voltage X‐rays generated by the linear accelerator (linac) and an amorphous silicon flat‐panel detector mounted on the linac to acquire and reconstruct the patient's volumetric image from a set of open‐field projection images acquired at different positions around the patient on the treatment couch. The MV‐CBCT images can provide 3D soft tissue and bony structure information. The system provides software tools for registration of the MV‐CBCT and planning CT images. Positional errors can then be estimated so that couch corrections can be made. The benefits of MV‐CBCT imaging are substantial and improve radiation treatment accuracy.

Recently, the MV‐CBCT system has been widely investigated in clinical applications. Many investigations have reported patient doses and image quality using the MV‐CBCT system in IGRT procedures.^21^, ^26^ However, these reports mainly focused on patient dose and image quality from MV‐CBCT images. Reports are sparse on the clinical use of the MV‐CBCT system with regard to alignment precision in the IGRT procedure. Morin et al.^(20)^ investigated the effect of different planning CT slice thicknesses on the localization accuracy of the MV‐CBCT system using an anthropomorphic head phantom. Their results indicated that the standard deviations of the differences between the applied shift and measured shift were 0.4 and 0.9 mm for 3D registration using 1 and 3 mm slice thickness CT scans, respectively.

The purpose of the present study was to evaluate the positioning accuracy of MV‐CBCT system using an anthropomorphic phantom, and investigate the impact of different CBCT imaging monitor units (MUs), reconstruction slice thicknesses, and planning CT slice thicknesses on positioning accuracy. Furthermore, patient CBCT images were used to retrospectively analyze initial setup errors and compare the setup precision of the MV‐CBCT system with different CBCT imaging MUs, reconstruction slice thicknesses, and planning CT slice thicknesses in head‐and‐neck patients.

## II. MATERIALS AND METHODS

### A. MV‐CBCT system

A commercially available MV‐CBCT system (MVision, Siemens Medical Solution, Concord, CA) was used in this study. This system is based on an ONCOR linac equipped with an amorphous silicon flat‐panel detector (AG9‐ES, Perkin Elmer Optoelectronics, Wiesbaden, Germany) adapted for MV photons. The area of effective measurement on the flat‐panel detector is $41\times 41{\;\text{cm}}^{2}$, and the flat‐panel detector has 1024×1024 detector elements with a pitch of 0.4 mm. The distance from the X‐ray source to the flat panel is 145 cm. The MV‐CBCT image acquisition procedure is similar to linac arc treatment. The linac gantry rotates in a continuous 200° arc (270°–110°, clockwise), delivering 6 MV photon energy and acquiring one portal image at each angle. The field width is fixed at 27.4 cm, and the field length is adjustable, with a maximum of 27.4 cm. The user creates imaging protocols by specifying the dose for MV‐CBCT acquisition (2–60 MU), reconstruction size (128×128, 256×256, or 512×512), and slice interval (1, 3, or 5 mm). The entire imaging system operates under Siemens SYNGOTM‐based COHERENCETM Therapist Workspace software, which communicates with the control console, linac, and patient database. The workspace software contains application software that allows the automatic acquisition of projection images, image reconstruction, planning CT to CBCT image registration, and couch position adjustment.

### B. Phantom and CT simulation localization

An anthropomorphic phantom (Chengdu Dosimetric Phantom, CPET Co. Ltd, Chengdu, China) was used for our study. The shape and anatomical structure of the phantom were similar to the human body. The equivalent error of the internal material as a substitute for the tissue or organs was less than 5%. The specification of this whole body phantom is described in ICRU Report 48.^(27)^

The phantom was immobilized in a vacuum bag and placed on the flat table of the planning CT scanner in a supine position. Using the laser positioning system in the CT room, skin marks were made with three lead markers at the head‐and‐neck region of the phantom. The head‐and‐neck region of the phantom was scanned using an AQUILION 16‐slice spiral CT (Toshiba, Otawara, Japan) with 1 and 3 mm slice thicknesses. The planning CT was done using helical scans. The matrix size of the CT scanner was 512×512. Two sequences of planning CT images of the phantom were acquired and transferred to the XiO treatment planning system (CMS, St. Louis, MO). The skin marks were used to determine the corresponding reference point within the phantom using a treatment planning system (TPS), and this reference point was labeled the planning treatment isocenter. The planning CT images of the phantom were transferred to the COHERENCE workstation. The imported planning CT images serve as the standard reference images of the MV‐CBCT system registration.

### C. Measurement of MV‐CBCT system positioning accuracy with the phantom

Five to 15 MU delivery is usually used in the clinic for MV‐CBCT image acquisition, with 5–8 MU delivery for the head‐and‐neck region and 8–15 MU delivery for the chest and pelvis regions. In the phantom measurements for the head‐and‐neck region, four scanning protocols of the MV‐CBCT were used: 5 MU delivery and 1 mm reconstruction slice thickness, 8 MU delivery and 1 mm reconstruction slice thickness, 5 MU delivery and 3 mm reconstruction slice thickness, and 8 MU delivery and 3 mm reconstruction slice thickness. The 256×256 reconstruction size and $27.4\times 27.4\;{\text{cm}}^{2}$ field size were used in all four scanning protocols. Two sets of planning CT images were used for the MVision system registration: planning CT images with 1 and 3 mm slice thicknesses.

**Figure 1:**
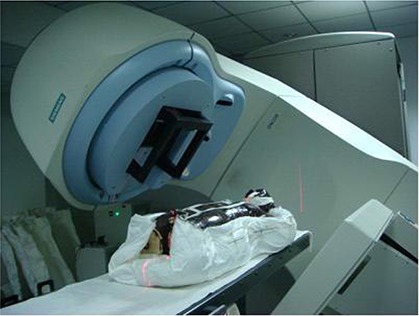
Anthropomorphic phantom measurement setup.

**Figure 2:**
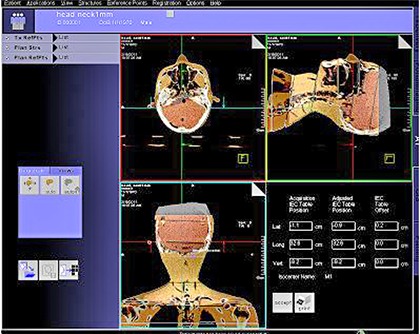
The MVision system automatically registered the CBCT images to the planning CT images and automatically performed shift measurement.

The phantom was placed on the treatment couch of the linac. The skin marks on the head‐and‐neck region were aligned using a laser positioning system so that the treatment center of the phantom coincided with the machine's isocenter (Fig. 1). A scanning protocol was then chosen to scan the head‐and‐neck region, and the MV‐CBCT system maximized a mutual information algorithm to determine the 3D shift between the phantom's treatment center and planning treatment isocenter at the isocenter position (Fig. 2). The shifts were then used to adjust the position of the treatment couch, and another CBCT scan was performed until the treatment center of the phantom coincided with the planning treatment isocenter at the isocenter position (the 3D shifts were all 0). This point was taken as the measurement origin.

The treatment center of the phantom was moved in six directions in 10 mm translational increments, including superior, inferior, left, right, anterior, and posterior using couch. The couch and lasers adjustment and alignment were made weekly, the tolerances for the couch and lasers were ≤ 1 mm. At each known position, all four scanning protocols were used, and the acquired CBCT images were than registered to the reference planning CT images of 1 mm and 3 mm slice thicknesses using the procedure described earlier. The deviation between the treatment center and planning treatment isocenter in the 3D directions were measured at the known position for different CBCT imaging MUs, reconstruction slice thicknesses, and planning CT slice thicknesses. The deviation in 3D directions was measured in 3 degrees of freedom as superior–inferior (SI), left–right (LR), and anterior–posterior (AP) translational errors.

The above process was repeated five times at different time intervals by the same group members. The data from all of the measurements were used to analyze the impact of different CBCT imaging MUs, reconstruction slice thicknesses, and planning CT slice thicknesses on the positioning accuracy of the MV‐CBCT system.

### D. MV‐CBCT system setup error in head‐and‐neck patients

Six patients with nasopharyngeal carcinoma who underwent IMRT treatment using a seven‐field step‐and‐shoot technique were chosen for this study. All the patients were immobilized in thermoplastic head‐and‐shoulder immobilization masks and treated in the supine position with a dose of 70 Gy in 35 fractions on the ONCOR linac using 6 MV X‐rays. For each patient, CT simulation localization was performed with 1 and 3 mm CT scanning slice thicknesses. The planning CT images were then used to plan IMRT treatment and for registration of the MV‐CBCT system.

Before IMRT treatment delivery, the skin marks on the patient were aligned using an in‐room laser positioning system. The CBCT scan was performed online using the MV‐CBCT with a 5 MU or 8 MU scanning protocol at 1 mm reconstruction slice thickness. The acquired CBCT images were then registered to 1 or 3 mm planning CT slice thickness to measure the patient setup error online. The reconstructed CBCT images with a 3 mm slice thickness were used offline and registered again to a 1 or 3 mm planning CT slice thickness to measure the patient setup error offline.

The MV‐CBCT system was used four times weekly for a total of 20 times for each patient in the full IMRT treatment course. Only 5 MU protocol was used for the verification of the patient's position in the last 10 fractions. Usually, some patients have tumor shrinkage or weight loss after 5 weeks IMRT treatment. These patients would require repeat head‐and‐shoulder thermoplastic mask making, panning CT imaging and replanning. Therefore, the design of this study was for the first 5 weeks in the treatment course. The 120 CBCT images acquired online and the 120 CBCT images reconstructed offline from six patients were used to retrospectively analyze the interfractional setup errors of the patients and investigate the impact of the different MV‐CBCT imaging MUs, reconstruction slice thicknesses, and planning CT slice thicknesses on the setup precision of the MV‐CBCT system.

### E. Statistical analysis

The 3D deviations are expressed as mean ± standard deviation. Analysis of variance (ANOVA) was used to compare differences between the imaging MUs, reconstruction slice thicknesses, and planning CT slice thicknesses. The statistical analyses were conducted using SPSS software, version 12.0 (Statistical Package for the Social Sciences, Chicago, IL). Values of *p* < 0.05 were considered statistically significant.

The random error (σ), systematic error (∑), and group systematic error (M) for the different CBCT imaging MUs, reconstruction slice thicknesses, and planning CT slice thicknesses were calculated using the definitions of van Herk.^(28)^ The random error (σ) was calculated using the root‐mean‐square of individual patient standard deviations (SDs). The distribution of systematic error (∑) was calculated using the SD of the individual mean errors.

## III. RESULTS

### A. Positioning accuracy of the MV‐CBCT system for the phantom

Table 1 shows the measured positioning deviation of the MV‐CBCT system between the treatment center and planning treatment isocenter using the phantom at known positions in 3D directions for the different CBCT imaging MUs, reconstruction slice thicknesses, and planning CT slice thicknesses. The results indicated no significant differences in the positioning accuracy between the 5 and 8 imaging MUs and between the 1 and 3 mm CBCT reconstruction slice thicknesses (p>0.05). While varying the planning CT slice thickness, the results indicated that the MV‐CBCT system positioning accuracy based on the 1 mm planning CT image registration was better than the positioning accuracy based on the 3 mm planning CT images. Significant differences were found in the SI direction in positioning accuracy between the 1 and 3 mm planning CT slice thicknesses (p<0.05). These results indicate that a thin‐layer planning CT registration may make the MV‐CBCT system positioning accuracy much higher. The largest measured mean deviation component and SD of the MVision in 3D directions were 1.3 and 1.0 mm, respectively, for different CBCT imaging MUs, reconstruction slice thicknesses, and planning CT slice thicknesses.

**Table 1:** Mean deviation of MVision positioning accuracy at six known positions for the different CBCT imaging MUs, reconstruction slice thicknesses, and planning CT slice thicknesses for the phantom study.

			*Deviation (mm)*		
*CBCT Imaging MUs* ^*a*^	*CBCT Slice Thickness* ^*b*^ *(mm)*	*Planning CT Slice Thickness* ^*c*^ *(mm)*	*SI*	*LR*	*AP*
5	1	1	0.6±0.5	0.4±0.3	0.5±0.0
		3	1.0±0.0	0.8±0.5	0.4±0.5
8	1	1	0.6±0.5	0.5±0.4	0.5±0.0
		3	1.3±0.5	1.0±0.7	0.3±0.5
5	3	1	0.5±0.4	0.5±0.4	0.5±0.0
		3	1.3±1.0	0.6±0.5	0.4±0.5
8	3	1	0.5±0.6	0.5±0.4	0.5±0.0
		3	1.0±0.8	0.5±0.4	0.3±0.5

a Compared with CBCT imaging MUs: $F_{\text{SI}}=0.0$, p=1.0; $F_{\text{LR}}=0.037$, p=0.849; $F_{\text{AP}}=0.261$, p=0.614.

^b^ Compared with CBCT slice thickness: $F_{\text{SI}}=0.090$, p=0.767; $F_{\text{LR}}=0.926$, p=0.346; $F_{\text{AP}}=0.0$, p=1.0

^c^ Compared with planning CT slice thickness: $F_{\text{SI}}=7.254$, p=0.013; $F_{\text{LR}}=0.037$, p=0.849; $F_{\text{AP}}=2.348$, p=0.139

### B. MV‐CBCT system setup errors in head‐and‐neck patients

Table 2 shows the estimated setup errors of the patients in 3D directions using the MV‐CBCT system at 5 and 8 imaging MUs and 1 and 3 mm CBCT slice thicknesses with 1 and 3 mm planning CT slice thicknesses as registration. The results indicated no significant differences in the setup errors between the 5 and 8 imaging MUs, 1 and 3 mm CBCT slice thicknesses, and 1 and 3 mm planning CT slice thicknesses in head‐and‐neck patients (p>0.05). The largest group systematic error (M), systematic error (∑), and random error (σ) were 0.6, 1.2, and 1.7 mm, respectively. The ∑ values were all larger than the M values in this study. This may be caused by motion of the bony anatomy with respect to the skin, incorrect placement of the skin markings, or changes in muscle tone.

**Table 2:** Estimates of group systematic error (M), systematic error (∑), and random error (σ) with different CBCT imaging MUs, reconstruction slice thicknesses, and planning CT slice thicknesses in six head‐and‐neck patients.

			*Setup Error (mm)*								
*CBCT Imaging MUs* ^*a*^	*CBCT Slice Thickness* ^*b*^ *(mm)*	*Planning CT Slice Thickness* ^*c*^ *(mm)*	*M*	*SI* ∑	σ	*M*	*LR* ∑	σ	*M*	*AP* ∑	σ
5	1	1	−0.1	0.6	1.7	0.1	0.5	1.0	0.0	0.3	1.2
		3	−0.6	0.8	1.3	−0.1	0.7	1.0	−0.3	0.6	0.8
8	1	1	−0.6	1.2	1.4	0.2	0.5	0.9	−0.2	0.9	1.2
		3	−0.6	1.2	1.5	0.0	0.6	1.1	−0.3	0.6	0.9
5	3	1	−0.1	0.6	1.7	0.1	0.5	1.0	0.1	0.3	1.2
		3	−0.5	0.7	1.3	−0.1	0.7	1.0	−0.2	0.5	0.9
8	3	1	−0.4	0.8	1.3	0.3	0.5	1.0	0.1	0.9	1.2
		3	−0.5	1.1	1.4	0.0	0.5	1.0	−0.3	0.6	0.9

^a^ Compared with CBCT imaging MUs: $F_{\text{SI}}=0.637$, p=0.430; $F_{\text{LR}}=0.712$, p=0.404; $F_{\text{AP}}=0.206$, p=0.653

^b^ Compared with CBCT slice thickness: $F_{\text{SI}}=0.0$, p=1.0; $F_{\text{LR}}=0.105$, p=0.747; $F_{\text{AP}}=0.526$, p=0.472

^c^ Compared with planning CT slice thickness: $F_{\text{SI}}=1.682$, p=0.202; $F_{\text{LR}}=0.826$, p=0.369; $F_{\text{AP}}=2.377$, p=0.131

## IV. DISCUSSION

When the system was commissioned, a lot of work was done using various phantoms to verify the system performance including the positioning accuracy of the system. The process of using the phantom studies in the manuscript was only a part of the process of commissioning of the system. The purpose of this study was for reasonably and effectively applying MV‐CBCT system and giving reasonable MV‐CBCT scan parameters and registration conditions in clinical setting.

The present study showed that the MV‐CBCT system had high positioning precision at the known positions when automatic registration was performed using an anthropomorphic phantom. This accuracy basically reflects the characteristics of the Siemens MV‐based IGRT system, with the MV‐CBCT imaging system and accelerator sharing one isocenter, which can improve the geometric precision of the CBCT scan. This advantage is also attributable to the precision of the registration software provided by the MV‐CBCT system.

The goal of our institute is to achieve good patient positioning using a minimal dose. This study showed that the positioning accuracy and setup precision of the MV‐CBCT system were not different between the 5 and 8 imaging MUs. This result illustrates the sufficiency of 5 MU delivery with the MV‐CBCT in head‐and‐neck patients using automatic alignment to verify the patient treatment position, which can reduce additional patient doses in the IGRT procedure. With regard to the imaging MUs, 5 MU delivery should not impact positioning accuracy because bony anatomy was used for this study.

Planning CT images are the benchmark reference images with which the MV‐CBCT system automatically registers the slice thickness and possibly influences the positioning process. The present study used an anthropomorphic phantom to evaluate the alignment process of the MV‐CBCT system using different planning CT slice thicknesses. A relatively larger deviation was found when using a 3 mm planning CT slice thickness compared with a 1 mm planning CT slice thickness. Morin et al.^(20)^ manually verified the precision of the system using an internally placed metal marker. The present results are consistent with the Morin study and indicate that planning thin‐layer CT scanning may make positioning more precise. However, the clinical measurements of setup errors in head‐and‐neck patients indicated no significant differences between the 1 and 3 mm slice thicknesses of the planning CT during the registration and alignment processes. These results may be attributable to the uncertainty of the setup error caused by therapists, laser misalignment, or the thickness of the skin markers. A 3 mm planning CT slice thickness has typically been used clinically in head‐and‐neck patients for treatment planning, registration, and alignment. Because of the inherent uncertainty of the setup error in radiotherapy, a thinner planning CT slice thickness used for registration and alignment did not significantly improve the setup accuracy of the MV‐CBCT system for the studied head‐and‐neck patients.

The MV‐CBCT reconstruction slice thickness may be another factor that influences the alignment process. The results indicated that the MV‐CBCT reconstruction slice thickness had no effect on the positioning accuracy for the phantom and head‐and‐neck patients. When the MV‐CBCT system used the 3 mm reconstruction slice thickness, the time of system image acquisition and registration was shortened by 24%, compared with the 1 mm reconstruction slice thickness (2.47 minutes).

Determining whether the MV‐CBCT system can meet the needs of IGRT in positioning accuracy is crucial, which is also a global concern of radiologists and oncologists. The present investigation of the positioning precision of the MV‐CBCT system using an anthropomorphic phantom and clinical patients has great clinical importance, even though this study was limited to head‐and‐neck phantom and patients.

In clinical practice, positioning errors are not only translational in 3D directions, but also in rotational directions. Because of the unavailability of a rotational error calculation function in the MV‐CBCT software, this study did not investigate the measurement and verification of rotational errors.

For organs like prostate, which have been documented to move substantially relative to bony anatomy, implanted fiducials (3–5 mm in length) are often preferred for day‐to‐day alignment. It is possible that the user may get different results based on the planning CT, as well as the reconstruction thickness MVCT. This is something which will be addressed in a future work, as the scope of the present study was limited to head‐and‐neck cases where use of bony anatomy is sufficient for day‐to‐day alignment purposes.

## V. CONCLUSIONS

The Siemens MV‐CBCT IGRT system is capable of achieving high positioning precision in the implementation of IGRT procedures. The positioning accuracy of the MV‐CBCT system is not significantly different between the 5 and 8 imaging MUs or between the 1 and 3 mm reconstruction slice thicknesses in head‐and‐neck patients. A thin planning CT slice thickness may achieve higher positioning precision with phantom measurements, but no significant difference was found in the setup precision of the MV‐CBCT system based on the 1 and 3 mm planning CT images registered in clinical practice. While use of lower 5 MUs help in reducing patient imaging dosage, use of 3 mm reconstruction slice thickness help in improving workflow efficiency, marginally.
